# Individual differences in treatment effects of internet-based cognitive behavioral therapy in primary care: a moderation analysis of a randomized clinical trial

**DOI:** 10.1186/s13722-025-00546-1

**Published:** 2025-02-14

**Authors:** Karin Hyland, Danilo Romero, Sven Andreasson, Anders Hammarberg, Erik Hedman-Lagerlöf, Magnus Johansson

**Affiliations:** 1 Centre for Psychiatry Research, Department of Clinical Neuroscience, Karolinska Institutet & Stockholm Health Care Services, Region Stockholm, Stockholm, Sweden; 2 Department of Global Public Health, Karolinska Institutet, Stockholm, Sweden; 3 Centre for Dependency Disorders, Stockholm Health Care Services, Region Stockholm, Stockholm, Sweden; 4 Division of Psychology, Department of Clinical Neuroscience, Karolinska Institutet, Stockholm, Sweden; 5 Gustavsberg University Primary Care Center, Region Stockholm, Stockholm, Sweden

**Keywords:** Alcohol dependence, Severity of dependence, Primary care, Internet-based cognitive behavioral therapy, Moderation analysis, Individual differences

## Abstract

**Background and aims:**

Little is known regarding predictors of outcome in treatment of alcohol dependence via the internet and in primary care. The aim of the present study was to investigate the role of socio-demographic and clinical factors for outcomes in internet-based cognitive behavioral treatment (ICBT) added to treatment as usual (TAU) for alcohol dependence in primary care.

**Design:**

Secondary analyses based on data from a randomized controlled trial in which participants were randomized to ICBT + TAU or to TAU only.

**Setting:**

The study was conducted in collaboration with 14 primary care centers in Stockholm, Sweden.

**Participants:**

The randomized trial included 264 adult primary care patients with alcohol dependence enrolled between September 2017 and November 2019.

**Interventions:**

Patients in the parent trial were randomized to ICBT that was added to TAU (n = 132) or to TAU only (n = 132). ICBT was a 12-week intervention based on motivational interviewing, relapse prevention and behavioral self-control training.

**Measures:**

Primary outcome was number of standard drinks last 30 days. Sociodemographic and clinical predictors were tested in separate models using linear mixed effects models.

**Findings:**

Severity of dependence, assessed by ICD-10 criteria for alcohol dependence, was the only predictor for changes in alcohol consumption and the only moderator of the effect of treatment. Participants with severe dependence showed a larger reduction in alcohol consumption between baseline and 3-months follow-up compared to participants with moderate dependence. The patients with moderate dependence continued to reduce their alcohol consumption between 3- and 12-months follow-up, while patients with severe dependence did not.

**Conclusions:**

Dependence severity predicted changes in alcohol consumption following treatment of alcohol dependence in primary care, with or without added ICBT. Dependence severity was also found to moderate the effect of treatment. The results suggest that treatment for both moderate and severe alcohol dependence is viable in primary care.

*Clinical trial registration: *The study was approved by the Regional Ethics Board in Stockholm, no. 2016/1367–31/2. The study protocol was published in Trials 30 December 2019. The trial identifier is ISRCTN69957414, available at http://www.isrctn.com, assigned 7 June 2018, retrospectively registered.

**Supplementary Information:**

The online version contains supplementary material available at 10.1186/s13722-025-00546-1.

## Introduction

Alcohol dependence is a highly prevalent condition globally in which only a minority, around 10–20 percent of affected individuals, are reached with treatment [1–3]. Barriers such as lack of awareness, shame and stigma often prevent individuals from seeking treatment [4–7]. Because alcohol contributes to other health conditions [8], affected individuals may still seek primary care for related health issues. Although opportunistic screening and brief interventions are effective in such circumstances [9], their implementation in routine care remains low [3, 10, 11], highlighting the need for alternative approaches. We recently conducted a randomized controlled trial investigating the incremental effect of internet-based cognitive behavioral therapy (ICBT) added to routine primary care for individuals with alcohol dependence [12]. Our results suggested substantial individual variability in treatment effects. To inform more effective treatment planning in primary care, increased knowledge about participant characteristics associated with individual variability in treatment effects (moderators) are needed. In this paper, we aimed to identify moderators of the added benefit of ICBT for treating alcohol dependence in primary care.

Primary care presents a valuable opportunity to discuss alcohol habits and offer treatment when indicated. Patients find it acceptable to discuss their alcohol habits with health care professionals when such discussions are relevant to their presenting health issues [13, 14]. Furthermore, primary care settings have been suggested to be perceived as less stigmatizing than specialized care [15, 16], while offering comparable outcomes to specialized care [17].

Despite evidence supporting the effectiveness of screening and brief intervention for hazardous and harmful alcohol use in primary care [9], their implementation in routine practice remains low [3, 10, 11], partly because general screening is rarely carried out in primary care [18]. This research-practice gap highlights the need for novel approaches for addressing alcohol dependence in primary care. Internet-based treatments have emerged as a promising strategy in this context. Their effectiveness in reducing alcohol consumption has strong meta-analytic support [19], demonstrating outcomes that are non-inferior to specialized services [20]. Internet-based interventions have been reported to reduce stigma and barriers to seeking treatment among individuals with alcohol dependence [21], [22, 23], and general practitioners have endorsed them as an attractive option to integrate into primary care services for this condition [24].

Building on this research, a recent randomized controlled trial (RCT) conducted by our team investigated whether internet-based cognitive behavioral therapy (ICBT) added to treatment-as-usual in primary care for individuals with alcohol dependence is more effective than treatment-as-usual alone [12]. The study found weak evidence for an incremental effect of ICBT in the intention-to-treat analysis but significant added benefits in the per-protocol analysis. These mixed findings suggest substantial individual variability in the incremental effects of ICBT. Such individual differences in treatment effect do not appear to be unique to this trial; indeed, a recent systematic review of models of care for the treatment of alcohol use disorder in primary care called for more research into factors associated with individual variability in treatment effects [25], commonly referred to as moderators [47]. Increased understanding about treatment moderators can inform more effective treatment planning for individuals with alcohol dependence and, more specifically in this context, support primary care clinicians in determining for whom adding ICBT is beneficial.

Internet-based treatments offer unique features—such as availability, flexibility and self-directed engagement—that may appeal differently to various socio-demographic and clinical subgroups, potentially leading to varied treatment outcomes. For example, the flexibility to access treatment content at any time [26] could allow those with demanding schedules and caregiving responsibilities to engage with treatment more effectively. Inversely, barriers such as limited digital literacy [27] may disproportionately discourage some subgroups from engaging with internet-based treatments. For instance, uptake and attitudes towards digital technology is lower and less positive among older adults [28], in spite of considerable heterogeneity within age groups [29]. However, importantly, the direction of the moderation effect can contradict researchers’ expectations, as shown by several empirical findings. A meta-analysis by Riper et al. [19] found that internet interventions targeting problem drinking were significantly more effective for male participants, those with lower education levels, and individuals over the age of fifty-five. Similarly, although not demonstrating treatment effect heterogeneity, a recent study comparing computer-based versus face-to-face brief advice for individuals with at-risk drinking, showed comparable outcomes for both interventions among participants with low level of education and unemployment [30]. Furthermore, while participants’ attitudes towards alcohol-targeted internet interventions may vary across subgroups, initial attitudes do not necessarily influence subsequent treatment response, as shown by a recent study [31]. Arguably, these somewhat unexpected findings highlight the need for research into treatment moderators that does not limit the agenda to confirmatory, hypothesis-testing studies but complements them with exploratory approaches. Moreover, little is known about clinical characteristics (e.g. severity of dependence) that potentially moderate the effectiveness of ICBT, further supporting the need for exploratory research to identify a range of potential moderators.

In summary, ICBT shows promise as a novel approach to broaden the reach of treatment for alcohol dependence in primary care, attractive to patients and providers alike. However, a recent trial suggested individual variability in treatment effects when ICBT was added to routine primary care, motivating research into the participant characteristics that could influence such variability. Increased understanding about which subgroups that benefit most from ICBT in complement to treatment-as-usual could inform clinicians’ treatment planning and patient-treatment matching, ultimately to increase remission from alcohol dependence. In the current study, we aimed to explore the moderation effects of a range of socio-demographic and clinical factors on the efficacy of adding internet-based treatment to treatment-as-usual for alcohol dependence in primary care.

## Methods

### Study design

This study was a secondary analysis of data from an RCT in which participants were randomized to ICBT added to TAU or to TAU only [12, 32]. The RCT and the secondary analyses described herein, were approved by the Regional Ethics Board in Stockholm, Dnr 2016/1367-31/2. All participants provided informed consent.

### Participants

The parent study included 264 participants recruited from 14 primary care centers in Stockholm, Sweden. Patients were randomized at a ratio of 1:1 to ICBT + TAU (n = 132) or to TAU only (n = 132). Potential participants were informed about the study at the primary care centers and signed up themselves via the study website by reading information about the study, providing their informed consent to participate and completing screening assessment. The eligibility criteria were > 18 years of age, three or more criteria for alcohol dependence according to the ICD-10 and > 6 points for women > 8 points for men for hazardous consumption according to the AUDIT. The exclusion criteria were serious mental illness, substance-use disorder other than alcohol and nicotine, need of specialized treatment in psychiatry or addiction care, cognitive impairment and lack of Swedish language skills. Trial participants had a mean age of 51 years, 56 percent were females and 44 percent were male. Most participants had university education (post high school), were employed, co-habiting, and had a moderate severity of dependence. Most participants (95%) were born in Europe. The follow-up rate at 3-months was 87% (230/264) and at 12-months 68% (180/264), with no difference in attrition rate between treatment groups. For a full account of the original trial´s design, procedures, and outcomes, see [12, 32].

### Procedure

TAU meant that patients were scheduled to their general practitioner (GP) who provided feedback on the assessments and biomarkers and made a treatment plan together with the patient based on current routines on treating alcohol problems at the respective primary care center. There are no clear guidelines for managing alcohol problems in Swedish primary care or for the training of GPs regarding this. All participating GPs, in both study arms, were given a one-hour update on the general management of alcohol dependence, including the use of pharmaceuticals, prior to the study. The ICBT program was focused on reducing alcohol use and was based on motivational interviewing, relapse prevention and behavioral self-control training. It was delivered as a self-help intervention with no therapist contact.

### Measures

#### Treatment outcome

In the present study we used the number of standard drinks consumed during the last 30 days, assessed with the Timeline Follow-Back method (TLFB, [33]), as the primary outcome measure. The definition of a standard drink followed Swedish guidelines, corresponding to 12 g of alcohol.

#### Variable selection

The selection of potential moderators was conducted prior to analysis and guided by the research team’s clinical background knowledge, informed by relevant literature, and involved discussions on the conceptual overlap between different measures. While a correlation matrix was calculated to assess the relationships between variables, with a threshold of *r* > 0.5 used as an additional criteria for considering exclusion [34], the primary basis for exclusion was clinical relevance and conceptual overlap. In order to avoid redundancy and multi-collinearity, several clinical predictors were excluded from the analyses, and some sociodemographic predictors dichotomized. The AUDIT measure, which covers alcohol consumption and dependence criteria [35], was excluded due to its overlaps with both dependence, as measured by ICD–10 criteria, and TLFB-derived consumption measures. Indeed, the AUDIT sum score exhibited moderate associations with both the number of ICD-10 criteria (*r* = 0.43, p < 0.001) and a TLFB-derived measure of binge drinking (*r* = 0.4, p < 0.001). Instead of using the combined AUDIT score, we opted to measure consumption and dependence separately to avoid conflation. Binge drinking and alcohol-free days were excluded due to overlap with the outcome measure, drinks last 30 days at baseline (*r* = 0.8 [p < 0.001] and *r* = − 0.64 [p < 0.001], respectively). EQ 5D-5L [36] was excluded due to high overlap (*r* = − 0.53, p < 0.001) with Hospital Anxiety and Depression Scale (HADS [48] total score, of which we opted for HADS due to its higher clinical relevance. The final sociodemographic predictors used were: age (centered), sex (female/male), relationship status (married/cohabiting versus living alone/widowed), education level (higher: > 12 years of education), and employment status (employed or not). The final clinical measures used were: Severity of alcohol dependence assessed by number of ICD-10 [37] criteria at baseline (moderate: 3–4, severe: 5–6 criteria), symptoms of anxiety and depression assessed with HADS total score at baseline, and being prescribed pharmacotherapy (acamprosate, disulfiram, naltrexone or nalmefene) during treatment. Baseline HADS exhibited a Cronbach’s alpha of 0.89 (95% Duhachek confidence interval: 0.87–0.91).

### Statistical analyses

These secondary analyses extend the analytical approach of the parent study [12], which modelled the change in outcome using linear mixed effects models. The analyses were conducted in accordance with the intention-to-treat principle, with missing data accounted for by restricted maximum likelihood estimations. Time was treated as a categorical variable due to non-uniformly spaced intervals and limited data points, with the 3-month follow-up serving as the reference. Only random intercepts were included. The original trial evaluation considered the effects of time, treatment, and their interaction on the outcome. In this secondary analysis, we expanded the model by including eight candidate predictors, each in a separate model. Thus, a total of eight models were fitted. The five socio-demographic and three clinical factors presented above were included as fixed effects, and each separate model included two- and three-way interaction terms for each combination of treatment, time and the individual predictor. The three-way interaction thus tested if there was a difference in effect over time between treatment groups as a function of the predictor. Such an effect can be referred to as a moderator. An alpha level of 0.05 was used to determine statistical significance. As this was an exploratory study aiming to identify, rather than confirm predictors, no adjustments for multiple comparisons were conducted. To compare performance of candidate predictor models with a reference model (which only included treatment, time and their interaction as fixed effects), we used the likelihood ratio test based on the maximum likelihood estimator. For absolute measures of goodness-of-fit, we calculated both marginal and conditional R2. Initially, our plan was to include all significant moderators in a single model, to investigate the effect of each significant predictor above and beyond the influence of other covariates. However, after fitting the eight individual models, we found that only one model yielded a significant three-way interaction among time, treatment, and a candidate predictor. Consequently, it was unnecessary to include multiple moderators in a single model. All analyses were conducted within the R (v. 4.2.3) statistical software environment [38], using the lme4 [39] and lmerTest [40] and performance [41] packages.

## Results

When analyzing predictors of outcome in linear mixed effects models with the number of standard drinks during the last 30 days regressed on time and treatment group, we found only one predictor—severity of dependence—that had significant effect on change in alcohol consumption. In this model (presented in Table 1 and described here) participants with moderate dependence in TAU was used as reference and the intercept (64.478, SE = 6.586) represent the value among these participants at 3-months follow-up which was used as the reference time-point. All participants reduced their alcohol consumption significantly between 0 and 3-months. There were no significant differences in alcohol consumption at 3-months between participants in ICBT + TAU with moderate dependence (ICBT: − 15.012, SE = 8.853, p = 0.091), among participants in TAU with severe dependence (ICD: − 10.569, SE = 10.338, p = 0.307) nor among participants in ICBT + TAU with severe dependence (ICBT*ICD: 21.595, SE = 15.21, p = 0.156).

**Table 1:** Linear mixed effects model with severity of dependence, treatment and time, as predictors of number of standard drinks during the last 30 days

(Intercept)	64.478 (SE = 6.586, p < 0.001)
Time 0–3m	22.561 (SE = 6.289, p < 0.001)
Time 3–12m	− 14.401 (SE = 6.954, p = 0.039)
ICBT	− 15.012 (SE = 8.853, p = 0.091)
Severe dependence	− 10.569 (SE = 10.338, p = 0.307)
Time 0–3m*ICBT	13.473 (SE = 8.45, p = 0.112)
Time 3–12m*ICBT	0.812 (SE = 9.369, p = 0.931)
Time 0–3m*severe dependence	28.905 (SE = 9.893, p = 0.004)
Time 3–12m*severe dependence	27.447 (SE = 10.922, p = 0.012)
ICBT*severe dependence	21.595 (SE = 15.21, p = 0.156)
Time 0–3m*ICBT*severe dependence	− 31.93 (SE = 14.557, p = 0.029)
Time 3–12m*ICBT*severe dependence	− 7.893 (SE = 16.53, p = 0.633)

Over time participants in TAU with moderate dependence changed their drinking significantly between baseline and 3-months (time_0–3 m*22.561, SE = 6.289, p < 0.001). This change was slightly bigger in ICBT + TAU with moderate dependence, but the difference between groups was non-significant (time_0–3 m*ICBT: 13.473, SE = 8.45, p = 0.1129). Between baseline and 3-months follow-up, participants with severe dependence showed a larger reduction in alcohol consumption compared to participants with moderate dependence in TAU (time 0–3m*ICD: 28.905, SE = 9.893, p = 0.004). The model also revealed a significant three-way interaction of dependence and ICBT + TAU on the change in alcohol consumption between baseline and 3-months follow-up (time 0–3m*ICBT*ICD: − 31.93, SE = 14.557, p = 0.029). This result implicates that the interaction effect of dependence severity on change in alcohol consumption between baseline and 3-months was different depending on group allocation *or* that the effect of treatment group on change in alcohol consumption between baseline and 3-months is different depending on severity of dependence. For detailed information on this three-way interaction, see Subgroup analysis below.

Between 3- and 12-months follow-up participants with moderate dependence in TAU reduced their drinking significantly (time 3-12m: − 14.401, SE = 6.954, p = 0.039). This reduction was not significantly different to participants with moderate dependence in ICBT + TAU (time 3–12m*ICBT: 0.812, SE = 9.369, p = 0.931). Participants with severe dependence in TAU increased their alcohol consumption between 3- and 12-months compared to those with moderate dependence (time 3–12*ICD: 27.447, SE = 10.922, p = 0.012). There was no significant three-way interaction of dependence and ICBT on the change in alcohol consumption between 3- and 12-months follow-up (time 3–12m*ICBT*ICD: − 7.893, SE = 16.53, p = 0.633).

Estimated unstandardized regression coefficients from the linear mixed effects model, Standard Errors (SE) within parentheses. Severe dependence was coded as 0 = moderate dependence (3–4 ICD-10 criteria), 1 = severe dependence (5–6 ICD-10 criteria). ICBT was coded as 0 = TAU, 1 = ICBT + TAU. Time 0-3m was coded as 0 = 3 months follow-up, 1 = Baseline. Time 3–12 was coded as 0 = 3 months follow-up, 1 = 12 months follow-up.

**Fig. 1:**
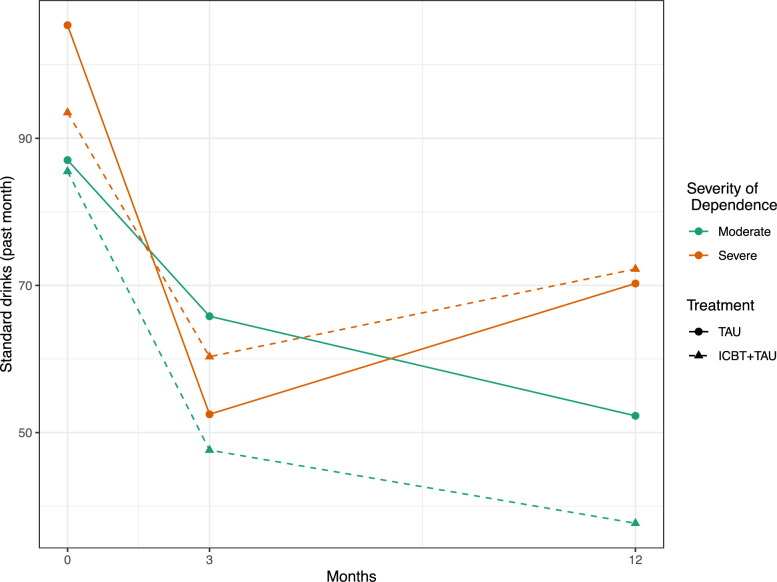
Observed mean number of standard drinks in the past month by treatment and severity of alcohol dependence, across time points (0, 3 and 12 months). Moderate dependence corresponds to 3–4 ICD-10 criteria, and severe dependence corresponds to 5–6 ICD-10 criteria. *ICBT* Internet-based Cognitive Behavioral Therapy, *ICD-10* Tenth revision of the International Classification of Diseases, *TAU* Treatment-As-Usual

To further understand the three-way interaction described above and the development in alcohol consumption over different time points, estimated means in different subgroups according to treatment and dependence severity were calculated and plotted in Fig. 1.

The only significant difference found in this analysis of subgroups was among participants in TAU. Participants in TAU reduced their drinking more between baseline and 3-months if they had severe vs moderate dependence at baseline. See Table 2 for details on the subgroup analysis.

**Table 2:** Subgroup analysis of differences in changes in standard drinks last 30 days between baseline and 3-months

Between baseline and 3-months	
TAU moderate vs. severe dependence	28.9 (SE = 9.89, p = 0.003)
ICBT + TAU moderate vs. severe dependence	− 3.03 (SE = 10.7, p = 0.777)
Moderate dependence TAU vs ICBT + TAU	− 18.5 (SE = 11.9, p = 0.119)
Severe dependence TAU vs ICBT + TAU	13.5 (SE = 8.45, p = 0.111)
Between 3 and 12-months	
TAU moderate vs. severe dependence	− 27.4 (SE = 10.9, p = 0.012)
ICBT + TAU moderate vs. severe dependence	− 19.6 (SE = 12.4, p = 0.115
Moderate dependence TAU vs ICBT + TAU	− 0.81 (SE = 9.37, p = 0.931)
Severe dependence TAU vs ICBT + TAU	7.08 (SE = 13.6, p = 0.603)

Based on linear mixed effects models with severity of dependence as predictor

A post-hoc analysis where mean baseline alcohol consumption was equalized was performed to check if the differences in TAU between participants with severe or moderate dependence could be explained by higher alcohol consumption at baseline. The significant differences in changed drinking between participants with severe compared to moderate dependence in the TAU group remained (time 0–3m*ICD: − 21.61, SE = 10.30, p = 0.037) when baseline alcohol consumption was equalized.

In the remaining prediction models, no significant three-way interactions or interaction effects with time were found. Higher age was related to a higher alcohol consumption while female sex high education and being employed status was related to a lower alcohol consumption, but with no significant effect on change in alcohol consumption over time or between treatment groups. Three-way interactions with the different predictors, ICBT and time are found in Table 3. Detailed information on all prediction models are found in Supplementary material.

**Table 3:** Three-way interactions from all tested prediction models

Predictor	0 to 3MFU		3 to 12MFU	
	Parameter	Coef.	Parameter	Coef.
Age	time 0–3m*ICBT*age	0.074 (SE = 0.531)	time 3–12m*ICBT*age	− 0.52 (SE = 0.602)
Sex	time 0–3m*ICBT*female	− 2.782 (SE = 13.913)	time 3–12m*ICBT*female	23.438 (SE = 15.556)
Relationship status	time 0–3m*ICBT*partner	− 4.281 (SE = 14.192)	time 3–12m*ICBT*partner	− 9.793 (SE = 15.953)
Education level	time 0–3m*ICBT* high education	2.64 (SE = 14.369)	time 3–12m*ICBT* high education	− 12.297 (SE = 16.102)
Working status	time 0–3m*ICBT* working	1.847 (SE = 16.422)	time 3–12m*ICBT*working	− 2.201 (SE = 18.143)
Dependence	time 0–3m*ICBT*severe	− 31.93* (SE = 14.557)	time 3–12m*ICBT*severe	− 7.893 (SE = 16.53)
Anxiety & depression	time 0–3m*ICBT*HADS	0.284 (SE = 0.966)	time 3–12m*ICBT*HADS	1.904 (SE = 1.064)
Pharmaco-therapy	time 0–3m*ICBT* medication	− 2.63 (SE = 36.17)	time 3–12m*ICBT*med	− 9.79 (SE = 16.30)

*p < 0.05 **p < 0.01 ***p < 0.001

## Discussion

The aim of this study was to investigate predictors of drinking for patients with alcohol dependence after treatment in primary care and with ICBT. These secondary analyses were based on data from a randomized controlled trial where ICBT was used as a complement to TAU and compared to TAU only. The only predictor that affected change in drinking over time and moderated outcome of treatment was degree of dependence.

Predictors in terms of age, sex, relationship status, education level, working status, symptoms of depression and anxiety or prescribed pharmacotherapy during treatment did not significantly affect changes in drinking. As reported in the parent trial [12], the participants were quite homogenous in terms of socio-demographics, which might explain why this study did not find any demographic factors that predicted drinking outcome. However, these socio-demographics might also be representative of treatment-seeking patients with alcohol dependence in primary care as they were similar to those observed in other studies conducted in that setting [17]. As also reported in the parent trial, many participants were prescribed pharmacotherapy and more participants in the TAU group got prescriptions compared to the ICBT + TAU group [12]. However, the analysis in this study could not find any significant effects on outcome of receiving prescriptions from their GP.

Both participants with severe and moderate alcohol dependence at baseline, reduced their drinking significantly between baseline and 3-months follow up. Participants with moderate dependence continued to reduce their drinking significantly between 3- and 12-months. Participants with severe dependence increased their drinking, but not significantly, between 3 and 12 months. The results indicate that the treatments offered via primary care in this trial may not be enough to help all participants with more severe dependence to continue or maintain changes in drinking after the treatment has ended. These results are in line with findings by Wallhed-Finn et al. [17]. All participants in this study had alcohol dependence and the results, similarly to the results in this present study, indicate that treatment in primary care might not be sufficient among individuals with more severe dependence.

The analysis of predictors also found a significant three-way interaction between time, treatment and dependence severity. This shows that getting access to ICBT or only getting TAU affected change in alcohol consumption differently if participants dependence was severe or if it was moderate. A subgroup analysis showed that participants in the TAU only group with severe dependence had a significantly larger reduction in alcohol consumption between baseline and 3-months follow-up, compared to participants with moderate dependence in TAU. The difference in changed drinking between participants with severe and moderate dependence in TAU could indicate that primary care treatment was more important or used differently by those with severe dependence. Other possible explanations to this finding could be that severe dependent participants, because they had experienced more alcohol related consequences, may have had more reasons to reduce alcohol consumption. Severely dependent participants in TAU also drank more at baseline and had increased room for improvement. But an analysis with equalized baseline drinking showed that this difference did not explain the significantly larger decrease in drinking among participants with severe alcohol dependence.

The participants with a moderate severity of dependence in the intervention group that had ICBT added to TAU showed a larger, but non-significant, reduction in alcohol consumption between baseline and 3-months follow-up compared to the control group that got TAU only. The decrease in drinking among moderately dependent individuals receiving ICBT continued after treatment. The subgroup analysis suggested a substantial effect where participants with moderately severe dependence in the ICBT + TAU group reduced their consumption by about 30 units per month at the 12-months follow-up compared to those with severe dependence in both groups. Behavioral self-control training was developed for problem drinkers with controlled drinking as a treatment goal [42]. The ICBT in this trial included several components from behavioral self-control training that can help moderately dependent drinkers identify with the program content. Users of another version of the ICBT-program described identification with the content as helpful [22].

Given a choice, the large group of individuals with alcohol dependence prefers treatment in primary care rather than in specialized care [14, 16]. Importantly, ICBT does not add substantially to the workload for GPs, who are struggling with time constraints and furthermore perceive a lack of competence to treat alcohol dependence [43, 44]. In automated ICBT the knowledge about how to change alcohol use and dependence is delivered through the program in the same way with each patient and is not dependent on clinician time or skill. A major challenge remains for practitioners: to raise questions about drinking that make sense to patients. Pragmatic case finding or targeted screening [45] has emerged as a possible alternative to general screening, which has failed to catch on in clinical practice [18, 46]. The parent trial demonstrates an alternative way where patients in primary care, interested in reducing their alcohol consumption, could raise the issue themselves by signing up to the project. General practitioners with experience of patients treated with ICBT were interviewed in a qualitative study [24]. The participating GPs perceived that availability of ICBT as a treatment to offer patients may enhance the likelihood to raise questions about alcohol. What the findings from the current study adds is that once identified, both moderately and severe alcohol dependent drinkers can successfully reduce their drinking through a brief intervention in primary care, and both TAU and ICBT are possible ways of offering alcohol interventions to patients. Assessing severity of dependence can serve as a practical tool for practitioners in primary care, where patients with 5 or 6 fulfilled criteria should be followed-up after the intervention and potentially be offered additional support or specialized treatment.

### Strengths and limitations

This secondary analysis is, to our knowledge, the first study to evaluate potential moderators of treatment effects in ICBT delivered as add-on to routine primary care. As the study was based on data from an RCT conducted in regular primary care. Data included information on several variables based on previous studies that might predict changes in alcohol consumption. The parent study had high follow-up rates compared to other studies of internet interventions. A limitation of this study was that the trial's power calculation was conducted with the primary research question in mind, specifically to detect a potential difference of *d* = 0.4 (effect size) between the treatment groups [12], rather than to analyze moderation. Consequently, while our sample size of *n* = 264 was sufficient for addressing the primary aim of the trial, it may be relatively small for exploring heterogeneity in treatment effects. This limitation restricts our ability to leverage comprehensive data-driven approaches, such as machine learning methods. While we recognize the limitations imposed by our existing data, we opted for a pragmatic approach that utilized available data to address our research questions. An interesting area for future research, when more data becomes available, is to leverage machine learning methods for exploring treatment effect heterogeneity. Outcomes and predictors used were mostly self-reported data and can have been influenced by social desirability or other factors that may lead to underreporting. Answering follow-up questions online can have reduced some of this risk. The trial participants were self-selected individuals that were homogenous in terms of socio-demographics and results therefore may not be generalizable to the untreated population with alcohol dependence, that might be less motivated to change and more demographically diverse.

## Conclusions

Treatment for both moderate and severe alcohol dependence is viable in primary care. Adding the offer of an internet intervention in primary care could help GPs raise questions about alcohol and also encourage patients to seek such treatment. This secondary analysis of treatment of alcohol dependence in primary care, with or without added ICBT, shows that dependence severity is a predictor of change in alcohol consumption. Dependence severity also moderated the effect of ICBT added to TAU and of TAU. The results suggest a need for monitoring patients with severe dependence after treatment to detect need for more care.

## Supplementary Information

Supplementary Material 1.

## Data Availability

The datasets analyzed during the current study and the web-based intervention are available from the last author on reasonable request.
