# Supplementary material for: Individual differences in treatment effects of internet-based cognitive behavioral therapy in primary care: a moderation analysis of a randomized clinical trial
Source: Addict Sci Clin Pract. 2025 Feb 14;20:17. doi: 10.1186/s13722-025-00546-1 (PMC11827356; doi:10.1186/s13722-025-00546-1)
Supplement: Supplementary file 1 — Supplementary Material 1. [file 13722_2025_546_MOESM1_ESM.docx]

Table S1. Linear mixed effects model with age, treatment and time, as predictors of number of standard drinks during the last 30 days.

|  | coef |
| --- | --- |
| (Intercept) | 61.944 (SE = 4.991, p < .001) |
| time 0-3m | 34.731 (SE = 4.922, p < .001) |
| time 3-12m | -3.327 (SE = 5.424, p = 0.54) |
| ICBT | -9.989 (SE = 7.034, p = 0.156) |
| age | 1.121 (SE = 0.364, p = 0.002) |
| time 0-3m*ICBT | 0.126 (SE = 6.937, p = 0.985) |
| time 3-12m*ICBT | -4.894 (SE = 7.787, p = 0.53) |
| time 0-3m*age | 0.342 (SE = 0.359, p = 0.341) |
| time 3-12m*age | 0.37 (SE = 0.403, p = 0.359) |
| ICBT*_cent | -0.662 (SE = 0.538, p = 0.22) |
| time 0-3m*ICBT*age | 0.074 (SE = 0.531, p = 0.889) |
| time 3-12m*ICBT*age | -0.52 (SE = 0.602, p = 0.388) |

Note: Estimated unstandardized regression coefficients from the linear mixed effects model, Standard Errors (SE) within parentheses. ICBT was coded as 0=TAU, 1=ICBT+TAU. Time_0-3m was coded as 0= 3 months follow-up, 1= Baseline. Time 3-12 was coded as 0= 3 months follow-up, 1= 12 months follow-up. Age was centered.

Table S2. Linear mixed effects model with sex, treatment and time, as predictors of number of standard drinks during the last 30 days.

| (Intercept) | 74.856 (SE = 7.409, p < .001) |
| --- | --- |
| time 0-3m | 37.096 (SE = 7.237, p < .001) |
| time 3-12m | 0.14 (SE = 7.861, p = 0.986) |
| ICBT | -13.198 (SE = 10.786, p = 0.222) |
| woman | -26.766 (SE = 10.055, p = 0.008) |
| time 0-3m*ICBT | 3.08 (SE = 10.533, p = 0.77) |
| time 3-12m*ICBT | -18.735 (SE = 11.738, p = 0.111) |
| time 0-3m* woman | -5.542 (SE = 9.817, p = 0.573) |
| time 3-12m*woman | -7.145 (SE = 10.824, p = 0.51) |
| ICBT*woman | 11.552 (SE = 14.255, p = 0.418) |
| time 0-3m*ICBT*woman | -2.782 (SE = 13.913, p = 0.842) |
| time 3-12m*ICBT*woman | 23.438 (SE = 15.556, p = 0.133) |

Note: Estimated unstandardized regression coefficients from the linear mixed effects model, Standard Errors (SE) within parentheses. Time_0-3m was coded as 0= 3 months follow-up, 1= Baseline. Time 3-12 was coded as 0= 3 months follow-up, 1= 12 months follow-up. Woman was coded 0= Man, 1= woman

Table S3. Linear mixed effects model with marital status, treatment and time, as predictors of number of standard drinks during the last 30 days.

| (Intercept) | 68.531 (SE = 8.321, p < .001) |
| --- | --- |
| time 0-3m | 22.626 (SE = 7.953, p = 0.005) |
| time 3-12m | -3.248 (SE = 9.046, p = 0.72) |
| ICBT | -5.337 (SE = 11.834, p = 0.652) |
| married | -12.645 (SE = 10.531, p = 0.23) |
| time 0-3m*ICBT | 3.542 (SE = 11.294, p = 0.754) |
| time 3-12m*ICBT | 0.509 (SE = 12.793, p = 0.968) |
| time 0-3m* married | 18.611 (SE = 10.057, p = 0.065) |
| time 3-12m* married | 0.196 (SE = 11.251, p = 0.986) |
| ICBT* married | -3.918 (SE = 14.875, p = 0.792) |
| time 0-3m*ICBT* married | -4.281 (SE = 14.192, p = 0.763) |
| time 3-12m*ICBT* married | -9.793 (SE = 15.953, p = 0.54) |

Note: Estimated unstandardized regression coefficients from the linear mixed effects model, Standard Errors (SE) within parentheses. Time_0-3m was coded as 0= 3 months follow-up, 1= Baseline. Time 3-12 was coded as 0= 3 months follow-up, 1= 12 months follow-up. Married was coded 0 = single or widowed, 1= married or cohabitating.

Table S4. Linear mixed effects model with education level, treatment and time, as predictors of number of standard drinks during the last 30 days. Higher education is > 12 years.

| (Intercept) | 73.284 (SE = 8.119, p < .001) |
| --- | --- |
| time 0-3m | 32.976 (SE = 7.848, p < .001) |
| time 3-12m | -11.793 (SE = 8.765, p = 0.179) |
| ICBT | -8.591 (SE = 11.844, p = 0.469) |
| Higher_education | -20.857 (SE = 10.41, p = 0.046) |
| time 0-3m*ICBT | -0.964 (SE = 11.447, p = 0.933) |
| time 3-12m*ICBT | 2.072 (SE = 12.88, p = 0.872) |
| time 0-3m*Higher_education | 2.438 (SE = 10.07, p = 0.809) |
| time 3-12m*Higher_education | 13.92 (SE = 11.17, p = 0.213) |
| ICBT*Higher_education | 2.608 (SE = 14.859, p = 0.861) |
| time 0-3m*ICBT* Higher_ education | 2.64 (SE = 14.369, p = 0.854) |
| time 3-12m*ICBT* Higher_ education | -12.297 (SE = 16.102, p = 0.445) |

Note: Estimated unstandardized regression coefficients from the linear mixed effects model, Standard Errors (SE) within parentheses. Time_0-3m was coded as 0= 3 months follow-up, 1= Baseline. Time 3-12 was coded as 0= 3 months follow-up, 1= 12 months follow-up. Higher education was coded as 0=

Table S5. Linear mixed effects model with employment, treatment and time, as predictors of number of standard drinks during the last 30 days.

| **working** | |
| --- | --- |
| (Intercept) | 80.302 (SE = 9.863, p < .001) |
| time 0-3m | 31.84 (SE = 9.545, p < .001) |
| time 3-12m | -11.494 (SE = 10.669, p = 0.282) |
| ICBT | -19.373 (SE = 14.884, p = 0.194) |
| working | -26.884 (SE = 11.511, p = 0.02) |
| time 0-3m*ICBT | -0.578 (SE = 14.389, p = 0.968) |
| time 3-12m*ICBT | -4.217 (SE = 15.829, p = 0.79) |
| time 0-3m*working | 3.308 (SE = 11.14, p = 0.767) |
| time 3-12m*working | 11.072 (SE = 12.4, p = 0.372) |
| ICBT*working | 16.414 (SE = 16.983, p = 0.334) |
| time 0-3m*ICBT*working | 1.847 (SE = 16.422, p = 0.911) |
| time 3-12m*ICBT*working | -2.201 (SE = 18.143, p = 0.903) |

Note: Estimated unstandardized regression coefficients from the linear mixed effects model, Standard Errors (SE) within parentheses. ICBT was coded as 0=TAU, 1=ICBT+TAU. Time_0-3m was coded as 0= 3 months follow-up, 1= Baseline. Time 3-12 was coded as 0= 3 months follow-up, 1= 12 months follow-up.

Table S6. Linear mixed effects model with dependence level, treatment and time, as predictors of number of standard drinks during the last 30 days.

| (Intercept) | 64.478 (SE = 6.586, p < .001) |
| --- | --- |
| time 0-3m | 22.561 (SE = 6.289, p < .001) |
| time 3-12m | -14.401 (SE = 6.954, p = 0.039) |
| ICBT | -15.012 (SE = 8.853, p = 0.091) |
| Severe_dependence | -10.569 (SE = 10.338, p = 0.307) |
| time 0-3m*ICBT | 13.473 (SE = 8.45, p = 0.112) |
| time 3-12m*ICBT | 0.812 (SE = 9.369, p = 0.931) |
| time 0-3m*Severe_dependence | 28.905 (SE = 9.893, p = 0.004) |
| time 3-12m* Severe_dependence | 27.447 (SE = 10.922, p = 0.012) |
| ICBT*Severe_dependence | 21.595 (SE = 15.21, p = 0.156) |
| time 0-3m*ICBT* Severe_dependence | -31.93 (SE = 14.557, p = 0.029) |
| time 3-12m*ICBT* Severe_dependence | -7.893 (SE = 16.53, p = 0.633) |

Note: Estimated unstandardized regression coefficients from the linear mixed effects model, Standard Errors (SE) within parentheses. ICD Severe_dependence was coded as 0=moderate dependence (3-4 ICD-10 criteria), 1= severe dependence (5-6 ICD-10 criteria). ICBT was coded as 0=TAU, 1=ICBT+TAU. Time_0-3m was coded as 0= 3 months follow-up, 1= Baseline. Time 3-12 was coded as 0= 3 months follow-up, 1= 12 months follow-up.

Table S7. Linear mixed effects model with symptoms of anxiety and depression, treatment and time, as predictors of number of standard drinks during the last 30 days. Symptoms of anxiety and depression was measured with the total score Hospital Anxiety and Depression Scale (HADS)

| (Intercept) | 60.137 (SE = 5.146, p < .001) |
| --- | --- |
| time 0-3m | 34.539 (SE = 4.932, p < .001) |
| time 3-12m | -4.306 (SE = 5.469, p = 0.432) |
| ICBT | -7.092 (SE = 7.23, p = 0.327) |
| anxiety/depression | -0.575 (SE = 0.846, p = 0.497) |
| time 0-3m*ICBT | 0.532 (SE = 6.926, p = 0.939) |
| time 3-12m*ICBT | -4.788 (SE = 7.743, p = 0.537) |
| time 0-3m*anxiety/depression | 0.332 (SE = 0.811, p = 0.682) |
| time 3-12m*anxiety/depression | -0.889 (SE = 0.871, p = 0.308) |
| ICBT*anxiety/depression | -0.17 (SE = 1.174, p = 0.885) |
| time 0-3m*ICBT*anxiety/depression | 0.084 (SE = 1.124, p = 0.941) |
| time 3-12m*ICBT*anxiety/depression | 1.722 (SE = 1.235, p = 0.164) |

Note: Estimated unstandardized regression coefficients from the linear mixed effects model, Standard Errors (SE) within parentheses. ICBT was coded as 0=TAU, 1=ICBT+TAU. Time_0-3m was coded as 0= 3 months follow-up, 1= Baseline. Time 3-12 was coded as 0= 3 months follow-up, 1= 12 months follow-up. Anxiety/depression was measured with the centered HADS total score

Table S8 County of birth

| Where were you born? |  |  |
| --- | --- | --- |
| Sweden | 227 | 85.98 % |
| Another Nordic country | 13 | 4.92 % |
| The rest of Europe | 10 | 3.79 % |
| Asia | 5 | 1.89 % |
| Central- or South America- | 5 | 1.89 % |
| Africa | 2 | 0.76 % |
| North America | 2 | 0.76 % |
